# Clinical Reproducibility of the Stone Volume Measurement: A “Kidney Stone Calculator” Study

**DOI:** 10.3390/jcm12196274

**Published:** 2023-09-28

**Authors:** Arthur Peyrottes, Marie Chicaud, Cyril Fourniol, Steeve Doizi, Marc-Olivier Timsit, Arnaud Méjean, Laurent Yonneau, Thierry Lebret, François Audenet, Olivier Traxer, Frederic Panthier

**Affiliations:** 1 GRC n°20, Groupe de Recherche Clinique Sur La Lithiase Urinaire, Hôpital Tenon, Sorbonne Université, 75020 Paris, France; arthur.peyrottes@aphp.fr (A.P.); marie.chicaud@hotmail.fr (M.C.); steeve.doizi@aphp.fr (S.D.); olivier.traxer@aphp.fr (O.T.); 2 Service D’Urologie, Hôpital Européen Georges Pompidou, AP-HP.Centre, Université Paris-Cité, 20 rue Leblanc, 75015 Paris, France; cyril.fourniol@gmail.com (C.F.); marc-olivier.timsit@aphp.fr (M.-O.T.); arnaud.mejean@aphp.fr (A.M.); francois.audenet@aphp.fr (F.A.); 3 Service D’Urologie, Hôpital Tenon, AP-HP, Sorbonne Université, 4 rue de la Chine, 75020 Paris, France; 4 PIMM Laboratory, UMR 8006 CNRS-Arts Et Métiers ParisTech, 151 bd de l’Hôpital, 75013 Paris, France; 5 Service d’Urologie, CHU de Limoges, 2 Avenue Martin Luther King, 87000 Limoges, France; 6 Service d’Urologie, Hôpital Foch-Université Paris Saclay-UVSQ, 40 rue Worth, 92150 Suresnes, France; l.yonneau@hopital-foch.com (L.Y.); t.lebret@hopital-foch.org (T.L.)

**Keywords:** Kidney Stone Calculator, renal stone, segmentation, volume, concordance

## Abstract

Background: An accurate estimation of the stone burden is the key factor for predicting retrograde intra-renal surgical outcomes. Volumetric calculations better stratify stone burden than linear measurements. We developed a free software to assess the stone volume and estimate the lithotrity duration according to 3D-segmented stone volumes, namely the Kidney Stone Calculator (KSC). The present study aimed to validate the KSC’s reproducibility in clinical cases evaluating its inter-observer and intra-observer correlations. Methods: Fifty patients that harbored renal stones were retrospectively selected from a prospective cohort. For each patient, three urologists with different experience levels in stone management made five measurements of the stone volume on non-contrast-enhanced computed tomography (NCCT) images using the KSC. Results: the overall inter-observer correlation (Kendall’s concordance coefficient) was 0.99 (*p* < 0.0001). All three paired analyses of the inter-observer reproducibility were superior to 0.8. The intra-observer variation coefficients varied from 4% to 6%, and Kendall’s intra-observer concordance coefficient was found to be superior to 0.98 (*p* < 0.0001) for each participant. Subgroup analyses showed that the segmentation of complex stones seems to be less reproductible. Conclusions: The Kidney Stone Calculator is a reliable tool for the stone burden estimation. Its extension for calculating the lithotrity duration is of major interest and could help the practitioner in surgical planning.

## 1. Introduction

Kidney stones’ prevalence has increased constantly (10.6%) and could concern 30% of the population by 2050 [1]. The interventional management of kidney stones is dominated via flexible ureteroscopy (fURS), extracorporeal shockwave lithotripsy (SWL), and percutaneous nephrolithotomy (PCNL) [2,3,4]. To define the appropriate surgical modality, current international guidelines refer to the location and stone size based on its maximum diameter (MD) in one dimension [3,4]. With the arrival of the MOSES^®^ technology and the new thulium fiber laser, fURS is gaining beyond its limits [5,6]. Considering only the MD for the stone burden estimation seems inaccurate, as the two other axes are not taken into account. This could influence surgical outcomes such as lithotripsy duration (LD) and operative time, which are key factors underlying retrograde intra-renal surgical complications [7]. As an increasing part of the surgical planning, the stone burden estimation seems primordial, and using a three-dimensional (3D) quantification could be of major interest. Several mathematical formulas have been proposed to approximate the stone volume, such as the spherical (4/3 × Π × radius^3^), Ackerman’s (0.6 × Π × radius^2^), or cumulative diameter (sum of the largest diameter of the stone in all planes) formulas [8]. However, kidney stones are rarely as plain as geometric shapes, and none of these equations have been validated [9].

The “Kidney Stone Calculator” (KSC) is an extension of 3DSlicer (5.0), a free software platform used for the 3D visualization and reconstruction of medical images [10]. It estimates the stone volume (SV), using 3D segmentation from the NICCT Digital Imaging and Communication in Medicine (DICOM) images, independently of its shapes and numbers [8]. The second purpose of the KSC is to estimate the LD of fURS based on the following variables: laser source and settings, laser fiber diameter, and stone composition [8]. A pilot study confirmed its accuracy for LD estimation [11]. However, regarding the SV estimation reproducibility, the study protocol included a small number of cases with only two clinical cases, in which the maximum inter-observer variation was observed (15%). Thus, the KSC lacks from a dedicated evaluation of its ability to measure the stone volume accurately and independently from the operator.

To validate these preliminary results, we aimed to evaluate the inter-observer reproducibility of the KSC in stone volume estimations among clinical cases. We further aimed to determine the intra-observer concordance and to identify the factors that influenced the KSC’s reproducibility.

## 2. Materials and Methods

### 2.1. Kidney Stone Calculator Software

The “Kidney Stone Calculator” segments kidney stones from the NCCT DICOM series. The user defines the density range that visually fits with the stone shape and limits, in manually bone window [12]. Hence, the KSC provides the number of voxels and the volume (mm^3^) through 3D segmentation on one side and the expected duration of lithotripsy on the other. A dedicated tutorial is available to estimate the stone burden (https//www.youtube.com/watch?v=pZLXHdfJtP0&t=5s, accessed on 1 September 2022).

### 2.2. Experimental Setup

Fifty eligible patients with kidney stones from the KSC multicentric database were used for this study [11]. This cohort included nothing but clinical cases. Each patient had at least one stone located in the upper urinary tract. Patients harboring ureteral stones were excluded from the study. Calculi were of various shapes and compositions and were either uni- or bi-lateral.

After a 10 min instruction on the KSC and the segmentation process, three investigators, i.e., a urologist in training (junior: less than 25 fURS procedures per year), a senior urologist with no expertise in the field of endourology (between 25 and 75 fURS procedures per year), and an expert endourologist (more than 100 fURS procedures per year), assessed the SV of these 50 cases using the KSC. For each case, all participants were asked to repeat the measurement five times. Each measure was blinded from the previous results and settings. Two hundred fifty data were gathered per practitioner, for a total of 750 measures. These evaluations were conducted in a full-blind mode.

The main objective of this study was to compare the estimated stone volumes among the three observers. Thus, we used the inter-observer reproducibility as the primary endpoint. The secondary endpoint was the intra-observer reproducibility. Three subgroup analyses were planned a priori based on the complexity (incomplete or complete staghorn stone versus “plain” stone), the homogeneity (more than 600 UH range), and the location (renal pelvis versus calyces) of the stones.

### 2.3. Statistical Analysis

Qualitative data were presented as percentages, and quantitative data were displayed as means and medians with interquartile ranges (Q1–Q3). The inter-observer reproducibility required Lin and Kendall concordance coefficient calculations (Lin and Kendall CCCs) and the Student’s *t*-test. The intra-observer reproducibility evaluation included Kendall’s coefficient of concordance and the coefficient of variation (CV). A numerical representation of the inter-observer reproducibility was also made according to the Bland and Altman method. Subgroup analyses required Mann–Whitney statistical tests. All statistical analyses required the use of Rstudio and GraphPad Prism. *p* values less than 0.05 were considered statistically significant.

## 3. Results

Table 1 shows the demographic characteristics of the population. The latter was composed of 24 men (48%) and 26 women (52%) with a median age of 48 yr (38–61). Eighty percent of the patients (40/50) had a history of symptomatic urolithiasis, while eighty-six percent (43/50) reported having had a previous interventional or surgical treatment (SWL, JJ stent insertion, fURS, or PCNL). The stones were mostly located in the inferior calix (38%). Fifteen cases were considered as complex and twenty-seven patients had homogenous calculi. The maximum stone diameter ranged from 3.8 to 59.8 mm for a median estimated stone volume of 923 mm^3^ (314–3032).

### 3.1. Inter-Observer Analysis

**Figure 1:**
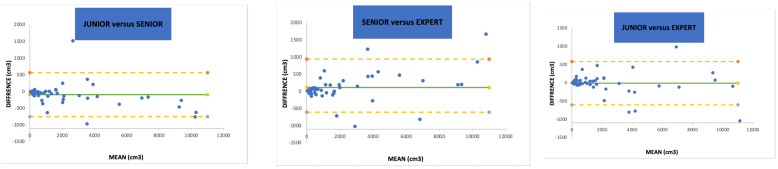
Bland and Altman representation of paired inter-observer reproducibility. SD = standard deviation. (--) = 95% intervals.

Table 2a shows the inter-observer reproducibility. The overall concordance between the three participants was 0.99 (*p* < 0.0001) using the Kendall CCC. When confronting two participants with a side-by-side comparison, there were no statistical differences in the SV measurements (*p* = 0.87, 0.85 and 0.98). The Lin CCCs were 0.99 (0.98–1) between the junior and senior urologists, 0.99 (0.98–0.99) between the senior urologist and the expert endourologist, and 0.99 (0.99–1) between the junior and expert endourologists. A numerical representation of the inter-observer reproducibility using the Bland and Altman method has been designed (Figure 1). The minimum standard deviation (SD) and repeatability coefficient (RC) were observed when comparing the junior endourologist to the expert endourologist as 302 and 593.47, respectively, whereas the maximum SD and RC were observed between the senior and expert urologists (SD 422 and RC 827.11).

### 3.2. Intra-Observer and Subgroup Analyses

The CV of the junior urologist, senior urologist, and expert endourologist were 6.3%, 4.2%, and 4.6%, respectively (Table 2b). The intra-operator concordances using the Kendall CCC were 0.98 for the junior urologist, 1 for the senior urologist, and 0.99 for the expert endourologist. Regarding the subgroup analyses (Table 2c), stone complexity was the only factor affecting reproducibility. The median CV was 11% for heterogenous calculi and 11.8% for homogenous ones (*p* = 0.51), whereas it was 11% for multiple calculi and 12% for unique stones (*p* = 0.78). Lastly, for the stones’ complexity criteria, the median CV was 13% for staghorn calculi and 8.1% for singletons (*p* = 0.02).

## 4. Discussion

### 4.1. Reproducibility and Agreement

In this study, we found a strong overall inter-observer correlation of 0.99. When comparing participants side by side, all concordance coefficient calculations were superior to 0.95, which is classified as an excellent agreement according to Partik’s benchmark scheme [13]. The intra-observer reproducibility was also reassuringly high, with the Kendall CCC ranging from 0.98 to 1. Finally, stone complexity was the only criterion predictive of significant changes in practitioners’ measurements, meaning that careful attention has to be paid when measuring the stone volume for complex stones. We emphasized that stone complexity was defined as a stone interesting at least one renal cavity and the renal pelvis or a group of cavities. A “Staghorn stone” refers to a complete occupation of the renal cavities by the stone, and it is not now as frequent as a few decades ago. Therefore, our definition of complex stones allows to integrate large stones with unusual shapes and filling a group of cavities that do not allow mobilization.

### 4.2. Stone Burden Evaluation

According to the international guidelines, the stone burden estimation is still based on the MD [2,3,4]. Recently, the SV evaluation was proposed to better stratify the stone burden [11]. As the interest of SV over MD or surface is still being debated within the urological community, the French urolithiasis guidelines [14] now suggest its use in cases of complex stones. In clinical practice, planar and volumetric measurements tend to show equal ability to predict the stone-free (SF) status, but volume estimations seem to be more accurate without repudiating the MD in most cases [15,16]. Ito et al. mentioned that the stone burden is better evaluated through the SV than the cumulative diameter (CD) formula or the surface area formula (maximum stone diameter × width × Π × 1/4). This is particularly validated when the CD is more than 20 mm or when there is more than three stones [17,18,19]. However, these authors used a mathematical formula to estimate the SV (length × width × height × Π × 1/6) rather than a segmentation process. Segmentation has originally been developed for other purposes than urinary stone volume estimation, and has proven its effectiveness under various carcinologic and non-carcinologic situations [20,21]. In the field of uro-nephrology, segmentation allows to gather precise anatomical information for diagnosis and surgical planning [22,23]. Hence, the segmentation process instinctively appears to be the most accurate method, justifying the use of the Kidney Stone Calculator. Other devices have been developed for this goal, but none of them aimed to predict the LD [24,25].

### 4.3. Clinical Implications

At a patient level, the Kidney Stone Calculator seems to improve the stone size appreciation and the choice of intervention. International guidelines dichotomize the surgical approach between external shockwave lithotripsy, flexible ureteroscopy, and percutaneous nephrolithotomy using the maximum diameter of the stone in one dimension [4]. However, the real burden of a spherical stone may be different to one measured using only the largest diameter. For example, a stone of 12 mm × 12 mm × 12 mm (904 mm^3^) is 3.5 times more substantial than one measuring 20 mm × 5 mm × 5 mm (262 mm^3^) when considering stones with a spherical shape (volume = 4/3 × Π × radius^3^). However, when considering only the maximum diameter, the second one is much wider than the first. An accurate estimation of the stone burden is needed for choosing the surgical modality, and the stone volume measured via segmentation might be the most appropriate method.

The second purpose of the KSC is to estimate the lithotripsy time during fURS. A longer operative time is known to be a risk factor for developing urosepsis after fURS, thus warranting the need for a good lithotripsy duration estimation [26]. The lithotripsy time depends on many variables, such as the stone composition, the laser source and settings, the fiber diameter, the use of ureteral sheath, and the location of the stone. Among them, the stone burden is surely the most important variable to pinpoint. The KSC was implemented with an algorithm allowing a reproducible estimation of the lithotripsy duration [11]. Hence, the surgeon using the KSC would be able to manage his laser settings and equipment in a more efficient way. He would also anticipate the procedure’s duration and the possible iterative sessions of fURS, enhancing his surgical program. Another approach to estimate the operative duration used the stone volume, density and number, the operator’s experience, sex, preoperative stenting, and ureteral sheath diameter [27]. Other models have been developed to estimate fURS outcomes. As one of them, the Resorlu–Unsal stone score has been introduced in 2012, and is currently validated both internally and externally for predicting the stone-free rate after fURS [28]. Lithotripsy duration estimation is a complex phenomenon of individual skillset and patient’s characteristics and being able to associate all these aspects would require a great amount of data. The first step in building such a model would be to understand the mechanism of stone ablation, which is still being debated.

Finally, using the KSC, the practitioner could also pre- and post-operatively evaluate himself, comparing the estimated lithotripsy duration to the effective one. For instance, it could be used as an education program for urologists in training to compare themselves to a “standard” and work on to master the learning curve.

### 4.4. Strengths and Limitations

The present study harbors several limits. First, we do not know the exact composition of the stones, since some participants did not perform their infrared spectrophotometry morpho-constitutional analysis and were lost during follow-up. It seems unlikely that this lack of information has altered the volume estimation, as the window used for the region of interest best fitted the stone characteristics. However, not knowing the stone chemical content may jeopardize the estimated lithotripsy duration. This limitation could be avoided using a prospective design. Secondly, we do not know the exact SV of each included case in the present study. This could lower the impact of our results, but the KSC’s ability to correctly measure an already known volume via segmentation has been demonstrated before [11]. Moreover, as the dusting technique is spreading, en-bloc extractions of stones are now less common. Consequently, we rarely dispose of the SV postoperatively [11]. Thirdly, the segmentation process is based on the sum of voxels gathered using a manual density scale on customized NCCT windows. It is well known that window settings impact stone size measurements, and the current evidence indicates that urinary calculi have to be analyzed in a bone window [29,30,31]. This aspect could be a measurement bias, but the intra- and inter-observer reproducibility results showed that practitioners defined constant NCCT windows and density scales. This finding is increased by the low required level of training to reach this reproducibility stage. The question of standardizing the window in which we analyze the stone volume remains as the automatization of the SV estimation using machine learning (ML) methods [32,33,34]. Finally, the aim of this study was to validate the accuracy of the KSC in estimating the stone volume in clinical cases. Fifty patients were randomly extracted from a prospective cohort for this purpose. This number could be debated, as we did not plan a number of patients to reach statistical significance. Five volume estimations were collected per patient and per practitioner for a total of 750 occurrences, which enhanced the power of the study. Drawing a parallel, the KSC’s pilot study included 26 patients, but demonstrated the feasibility of LD estimation [11]. Therefore, further evaluations are needed, including automatization of the segmentation process.

## 5. Conclusions

The stone volume is increasingly used over linear measures for stone burden estimation. The Kidney Stone Calculator is a free software tool that allows for the 3D segmentation of renal calculi and predict the lithotrity time. The present study shows that the KSC is a valid, independent, and reproductible device for estimating the stone volume. Having such a reliable tool is of major interest for upcoming urologists, as it will help train their surgical skills and enhance their surgical planning.

## Figures and Tables

**Table 1:** Demographic characteristics of the population.

Variables			Value
Patients			50
Age			48 (38–61)
Gender	Female		26 (52%)
	Male		24 (48%)
Stone history	Renal colic		40 (80%)
	Previous surgical procedure		43 (86%)
	Anatomical urinary tract abnormality		14 (28%)
Stone characteristics	Side	Left	27 (54%)
		Right	23 (46%)
	Number of calculi	1	24 (48%)
		2 or 3	18 (36%)
		>3	8 (16%)
		Complex	15 (30%)
	Localization	Lower pole	19 (38%)
		Renal pelvic	15 (30%)
		Other	16 (32%)
	Density, median (UH)		1100 (716–1546)
	Density (UH)	>1000 UH	31 (62%)
	Maximum diameter, median (mm)		15 (10–23)
	Segmented stone burden, median (mm^3^)		923 (314–3032)
	Homogeneous calculi		27 (54%)

**Table 2:** (**a**) Inter-observer reproducibility. CCC: concordance correlation coefficient; CI: 95% confidence interval. (**b**) Stone volume per patient and per practitioner and intra-observer reproducibility. (**c**) Median coefficients of variation through subgroup analyses.

(a)			
**Paired concordance**		**Junior versus senior urologist**	
**Lin CCC (CI)**		0.99 (0.98–1)	
Student’s *t*-test (*p*)		0.87	
**Paired concordance**		**Senior urologist versus expert endourologist**	
Lin CCC (CI)		0.99 (0.98–0.99)	
Student’s *t*-test (*p*)		0.85	
**Paired concordance**		**Junior versus expert endourologist**	
Lin CCC (CI)		0.99 (0.99–1)	
Student’s *t*-test (*p*)		0.98	
**Overall concordance (Kendall CCC)**		0.99 (*p* < 0.0001)	
(**b**)			
**Case number**	**Median volume (mm^3^) and Q1–Q3**		
	**Junior urologist**	**Senior urologist**	**Expert endourologist**
1	1720 (1675–1787)	1664 (1645–1691)	1609 (1598–1680)
2	1210 (1177–1216)	1209 (1196–1239)	1165 (1155–1191)
3	92 (92–96)	63.16 (59.31–63.63)	83 (82–90)
4	681 (672–684)	636.5 (632.8–642)	706 (630–710)
5	255 (249–265)	314.4 (312.1–320,5)	244 (225–248)
6	154 (141–159)	169.9 (161–170.8)	121 (119–178)
7	2184 (2154–2198)	1857 (1817–1970)	2049 (2023–2049)
8	190 (190–196)	204 (201–211)	178 (165–181)
9	648 (640–654)	604 (571.7–617.8)	664 (640–680)
10	472 (468–493)	424.1 (422.6–468)	530 (528–547)
11	1605 (1557–1610)	1643 (1600–1660)	1649 (1616–1706)
12	7404 (7311–7567)	7238 (7100–7360)	6423 (6423–6679)
13	7040 (6582–7047)	6844 (6815–6896)	7156 (7003–7178)
14	1903 (1900–1926)	3414 (3345–3518)	2391 (2377–2408)
15	987 (984–1034)	988 (921–994)	856 (824–856)
16	3114 (3059–3132)	2988 (2921–2994)	3132 (3132–3154)
17	177 (168–183)	176 (175–178)	120 (112–120)
18	312 (308–339)	315 (314–319)	215 (213–242)
19	1913 (1905–1980)	2161 (2129–2203)	1440 (1440–1440)
20	92 (83–96)	82 (81–82)	80 (77–84)
21	2180 (2173–2218)	1942 (1887–1983)	2057 (2048–2059)
22	983 (932–1009)	613 (565–624)	1006 (2048–2059)
23	3371 (3313–3469)	3731 (3731–3811)	4178 (4178–4178)
24	31 (30–33)	26 (25–27)	34 (25–34)
25	4016 (3990–4017)	3047 (3024–3052)	4276 (4276–4276)
26	9518 (9449–9521)	9247 (9167–9263)	9446 (9430–9459)
27	901 (897–948)	858 (828–864)	886 (886–886)
28	375 (399–399)	343 (339–351)	442 (395–444)
29	385 (384–399)	356 (340–357)	211 (211–211)
30	499 (485–503)	495 (482–498)	463 (463–497)
31	4257 (4239–4265)	4105 (4028–4106)	3830 (3581–4063)
32	128 (126–134)	123 (121–125)	162 (161–162)
33	5733 (5681–5740)	5352 (5305–5366)	5823 (5822–5826)
34	1378 (1354–1399)	1312 (1312–1332)	1497 (1497–1504)
35	346 (313–355)	216 (216–224)	261 (261–261)
36	1393 (1235–1696)	756 (755–758)	1352 (1346–1372)
37	873 (808–880)	604 (599–627)	510 (489–550)
38	252 (241–254)	226 (224–228)	254 (250–260)
39	297 (288–301)	294 (277–323)	361 (361–362)
40	9485 (9473–9499)	9021 (8707–9069)	9212 (9212–9212)
41	10,606 (10,337–10,624)	9982 (9924–10,011)	11,652 (11,652–11,652)
42	3805 (3723–3892)	4015 (4007–4136)	4581 (4581–4756)
43	249 (230–276)	152 (145–159)	234 (231–240)
44	266 (263–271)	240 (234–241)	273 (269–284)
45	1567 (1460–1786)	1621 (1494–1660)	1512 (1470–1514)
46	1187 (1037–1233)	1042 (1019–1076)	1234 (1208–1234)
47	2172 (2169–2200)	2033 (1019–1076)	2343 (2300–2412)
48	3671 (3660–3690)	3464 (3448–3493)	3897 (3578–4001)
49	10,621 (10,599–10,647)	9858 (9849–9916)	10,717 (10,717–10,717)
50	593 (559–598)	470 (461–485)	530 (524–532)
**Coefficient of variation (%)**	6.3%	4.2%	4.6%
**Intra-operator concordance (Kendall CCC)**	**0.98** (*p* < 0.0001)	**1** (*p* < 0.0001)	**0.99** (*p* < 0.0001)
(**c**)			
	**Homogenous**	**Heterogenous**	***p*-value (Mann–Whitney test)**
**Median coefficient of variation (%)**	11.8%	11%	0.51
	**Complex**	**Non-complex**	***p*-value (Mann–Whitney test)**
**Median coefficient of variation (%)**	13%	8.1%	0.02
	**Unique**	**Multiple**	***p*-value (Mann–Whitney test)**
**Median coefficient of variation (%)**	12.1%	11%	0.78

## Data Availability

Not applicable.
